# The Safety and Clinical Validity of Endoscopic Submucosal Dissection for Early Gastric Cancer in Patients Aged More Than 85 Years

**DOI:** 10.3390/cancers14143311

**Published:** 2022-07-07

**Authors:** Takaaki Yoshikawa, Atsushi Yamauchi, Ryuhei Hamasaki, Yuki Mori, Kazuki Osawa, Ryo Ito, Yuya Kawai, Souta Nakagami, Shunjiro Azuma, Toshihiro Morita, Kenshiro Hirohashi, Katsutoshi Kuriyama, Ken Takahashi, Tadayuki Kou, Hiroshi Kondoh, Shujiro Yazumi

**Affiliations:** 1 Department of Gastroenterology and Hepatology, Kitano Hospital, Tazuke Kofukai Medical Research Institute, 2-4-20, Ohgimachi, Kita-ku, Osaka 530-8480, Japan; a-yamauchi@kitano-hp.or.jp (A.Y.); hr.3315@noe.saiseikai.or.jp (R.H.); yuuki-mori@kitano-hp.or.jp (Y.M.); k-oosawa@kitano-hp.or.jp (K.O.); ryou-itou@kitano-hp.or.jp (R.I.); yuuya-kawai@kitano-hp.or.jp (Y.K.); s-nakagami@kitano-hp.or.jp (S.N.); s-azuma@kitano-hp.or.jp (S.A.); toshihiro-morita@kitano-hp.or.jp (T.M.); k-hirohashi@kitano-hp.or.jp (K.H.); k-kuriyama@kitano-hp.or.jp (K.K.); ken-takahashi@kitano-hp.or.jp (K.T.); t-kou@kitano-hp.or.jp (T.K.); s-yazumi@kitano-hp.or.jp (S.Y.); 2 Department of Gastroenterology and Hepatology, Noe Saiseikai Hospital, 1-3-25, Furuichi, Jyoto-ku, Osaka 536-0001, Japan; 3 Department of Geriatric Unit, Graduate School of Medicine, Kyoto University, 54 Shogoin-Kawahara-cho, Sakyo-ku, Kyoto 606-8507, Japan; hkondoh@kuhp.kyoto-u.ac.jp

**Keywords:** early gastric cancer, endoscopic submucosal dissection, elderly patients, overall survival, prognosis

## Abstract

**Simple Summary:**

In this study, we elucidated whether endoscopic submucosal dissection for early gastric cancer is safe and feasible in very old patients. We compared the characteristics and outcomes of patients aged ≥85 years with those of other patients. We found no significant differences in the incidence of adverse events between patients ≥85 years of age and other patients. However, the overall survival of patients aged ≥85 years was significantly lower than that of other patients. We found that poor nutritional status was correlated with poor prognosis in patients aged ≥85 years. Therefore, we conclude that endoscopic submucosal dissection for early gastric cancer is safe and valid for patients aged ≥85 years. However, the indications should be carefully decided because it is difficult to estimate the survival benefits of endoscopic submucosal dissection for early gastric cancer in patients aged ≥85 years, especially those with poor nutritional status.

**Abstract:**

Endoscopic submucosal dissection (ESD) is a safe and minimally invasive method for the treatment of early gastric cancer (EGC). However, whether ESD for EGC is also safe and feasible in patients aged ≥85 years is unclear. The patients enrolled in this study were divided into three groups: age ≥85 years (44 patients, 49 lesions), age 65–84 years (624 patients, 687 lesions), and age ≤64 years (162 patients, 174 lesions). We evaluated the incidence of adverse events (AEs) and overall survival (OS) and disease-specific survival (DSS). We analyzed the factors that had a significant impact on the prognosis of patients aged ≥85 years. No significant differences were found in the incidence of AEs among the three groups (*p* = 0.612). The OS was significantly lower in patients aged ≥85 years (*p* < 0.001). Conversely, DSS was not significantly worse in patients aged ≥85 years (*p* = 0.100). The poor Geriatric Nutritional Risk Index correlated with poor prognosis in patients aged ≥85 years (*p* < 0.001). ESD is a safe and valid treatment for EGC in patients aged ≥85 years. However, the indications should be carefully decided because it is difficult to estimate the survival contribution of ESD for EGC in patients aged ≥85 years, especially in those with poor nutritional status.

## 1. Introduction

Gastric adenocarcinoma is the sixth most common type of cancer worldwide and the third most common cause of cancer-related deaths [1]. Because surgical gastrectomy potentially impairs the quality of life of patients [2], minimally invasive treatment is needed, especially for early gastric cancer (EGC). Endoscopic submucosal dissection (ESD), an established method for endoscopic resection, is widely accepted worldwide. The use of ESD rapidly became widespread because it is safer and less invasive than conventional gastrectomy with radical lymphadenectomy [3].

According to the World Health Organization, the life expectancy of humans is increasing each year, and Japan is one of the countries with the longest life expectancy worldwide (81.5 years for men and 86.9 years for women in 2019). Since many EGCs are found among very elderly patients, the chance of undergoing ESD for EGC is high in this population. Several studies have reported the outcomes of ESD for EGC in elderly patients aged ≥75 years [4,5,6] or ≥80 years [7,8,9,10]. However, few studies have reported ESD for EGC in patients aged ≥85 years [11,12]. Because ESD for EGC has the potential to harm very elderly patients because of adverse events (AEs) such as aspiration pneumonia [6,8,13], it is quite important to elucidate whether AEs for very elderly patients are critical, whether very elderly patients can overcome AEs, and whether there is an aging border regarding the advantages of ESD for EGC.

In general, if ESD results in non-curative resection, additional gastrectomy should be recommended [14]. However, age is a potential risk factor for morbidity (10.1–19.4%), although the mortality rate (0–2.2%) does not significantly differ between the elderly and the non-elderly patients [15,16,17,18,19]. Other reports have shown that myocardial infarction and diabetes are risk factors for increased mortality after gastrectomy [20,21]. In addition, attention should be paid to the negative impact of gastrectomy on the quality of life of elderly patients [22,23]. Moreover, according to the latest Japanese gastric cancer treatment guidelines, close observation for some non-curative resected EGC is considered an option [24]. The scoring system for curability suggests that the risk of lymph node metastasis in some populations of non-curative resected EGC is very low [25]. Therefore, it is important to determine whether additional gastrectomy has prognostic benefits for patients aged ≥85 years, similar to those for patients aged < 85 years.

Therefore, we used historical cohort data to evaluate the safety and clinical impact of gastric ESD for EGC in patients aged ≥85 years. We hypothesized that similar to patients aged < 85 years, patients aged ≥85 years also underwent safe ESD. Furthermore, we evaluated the prognosis of ESD for EGC in patients aged ≥85 years compared to that in patients aged < 85 years. Finally, we analyzed the factors that had an impact on the prognosis of ESD for EGC in patients aged ≥85 years.

## 2. Patients and Methods

### 2.1. Patients

This historical cohort study was conducted at a single institution. We consecutively enrolled 939 patients (1019 lesions) who underwent ESD at Kitano Hospital (Osaka, Japan) between January 2006 and December 2020. The patients were classified into three groups: ≥85 years, 65–84 years, and <65 years. The clinical data of patients were collected from medical interviews, medical records, endoscopic reports, and pathological reports. The status of *Helicobacter pylori* infection could not be investigated because of missing data. In the cases of metachronous lesions, we counted the ESD sessions of the same patient separately. In contrast, patients with several lesions resected in one session were counted as one patient. If several lesions were resected in one piece, some indicators (e.g., operation time) were used only for the most advanced lesion. In contrast, when lesions were resected in separate pieces in one session, we counted each lesion independently.

The inclusion criterion was ESD for gastric adenocarcinoma including metachronous lesions. Procedure discontinuation because of perforation (*n* = 3) and muscular invasion (*n* = 1) were included in the analysis of patient characteristics and safety of ESD but were excluded from the pathological analysis.

The exclusion criteria were as follows: ESD for adenoma (*n* = 85), neuroendocrine tumor (*n* = 2), gastrointestinal stromal tumor (*n* = 1), or hyperplastic polyp (*n* = 1); no lesion (*n* = 6); laparoscopy and endoscopy cooperative surgery (*n* = 5); pre-cutting endoscopic mucosal resection (*n* = 1); hybrid ESD (*n* = 5); recurrence after pre-endoscopic treatment (*n* = 2); and other active malignancy (*n* = 1). We assessed the patients’ characteristics, including comorbidities before ESD, and mostly excluded bedridden patients, those with dementia, or patients with severe impairment in ADL. Therefore, six patients aged ≥85 years did not undergo ESD after the detection of EGC because of poor performance status (PS), comorbidities, and patient refusal. These patients could not be followed up and analyzed.

### 2.2. ESD Procedure

We carefully examined the patients’ EGD and computed tomography (CT) findings before performing ESD, and we strictly followed the Japanese gastric cancer treatment guidelines at that time [24,26,27]. Moreover, we assessed the patients’ characteristics, including comorbidities, and decided the indication for ESD after a consultation with other departments and a discussion at our department, if needed. Written informed consent was obtained from all patients. ESD was performed with an insulated tip (IT) knife (2006–2007) (KD-610L, Olympus, Tokyo, Japan), an IT knife 2 (2007–2012, mainly) (KD-611L, Olympus), a DualKnife (2012–2015) (KD-650L, Olympus), a DualKnife J (2015–2020, mainly) (KD-655L, Olympus), a flush knife (DK2620JI-B25; Fujifilm Medical, Tokyo, Japan), a hook knife (KD-620QR, Olympus), and a needle knife (KD-1L-1, Olympus). A solution consisting of 0.4% sodium hyaluronate (MucoUp; Boston Scientific, Marlborough, MA, USA) and glycerol (Hisiceol; Nipro, Osaka, Japan) supplemented with epinephrine was injected into the submucosa using a 25-G injection needle (01961; Top Corp., Tokyo, Japan) or a DualKnife J. An overtube (16403, Top Corp.) was regularly used. Hemostatic forceps (Coagrasper, FD410LR, Olympus) were used for prophylactic coagulation of blood vessels and for hemostasis for intraoperative bleeding. ICC200 (2006–2015) (Erbe Elektromedizin GmbH, Tübingen, Germany), VIO200D (2015–2018) (Erbe Elektromedizin GmbH) or VIO3 (2018–2020) (Erbe Elektromedizin GmbH) were used as the high-frequency generators. All patients were sedated with midazolam, propofol, or dexmedetomidine combined with pentazocine.

Second-look endoscopy was not regularly performed. With respect to the management of antithrombotic agents, we strictly followed the existing Japan Gastroenterological Endoscopy Society guidelines at that time [28,29].

### 2.3. Pathological Characteristics

In terms of tumor location, the upper (U), middle (M), and lower (L) portions of the stomach were defined according to the third English edition of the Japanese Classification of Gastric Cancer [30]. For pathological evaluation, resected specimens were sliced into 2-mm sections. With respect to histology, the differentiated type included well-differentiated and moderately differentiated adenocarcinoma and papillary adenocarcinoma. The undifferentiated type included poorly differentiated adenocarcinoma, signet-ring cell carcinoma, and mucinous adenocarcinoma. If the histological type was a mix of differentiated and undifferentiated types, we classified the lesion according to the predominant histological type.

### 2.4. Short-Term Outcomes

We evaluated en bloc resections, curative resections, operation time, and days of hospitalization as short-term outcomes. Curability was assessed in accordance with the eCura system, as defined in the second edition of guidelines of the Japanese Gastroenterological Endoscopy Association [14]. eCura A and B were defined as curative resections, whereas eCura C-1 and C-2 were defined as non-curative resections. Operation time was defined as the period between the first cut of mucosal dissection and the complete separation of a lesion from the stomach.

### 2.5. Adverse Events

Postoperative bleeding was defined as the occurrence of hematemesis or melena and a ≥2.0 g/dL decrease in hemoglobin level, and bleeding from a post-ESD ulcer was confirmed using EGD. Perforation was defined as the detection of an obvious defect in the muscular layer caused by the ESD procedure or the detection of free air by CT after ESD. Stricture was defined as the occurrence of food intake inability, and food retention in the stomach due to post-ESD scars was confirmed with EGD. Aspiration pneumonia was defined as the development of respiratory symptoms and fever ≥ 38°C.

### 2.6. Long-Term Outcomes and Follow-Up

The first postoperative EGD was performed within 3 months after ESD. The patients were followed up with physical examination, blood tests, EGD, and abdominal ultrasound or CT every year for at least 5 years consecutively. The follow-up period was calculated from the day of the ESD procedure to the date of death or the last confirmation of survival. The causes of death and the last confirmation of survival were confirmed through a telephone interview or a review of medical records. In cases of non-curative resection, additional gastrectomy was cooperatively decided by the patients and the physicians in charge after understanding the recurrence and perioperative risks.

### 2.7. Statistics

Values are presented as mean ± standard deviation or median (range [minimum–maximum]) values. The Kruskal–Wallis test was used for grouped continuous data not meeting the Gaussian distribution. The chi-square test was used for statistical analysis of categorical data. Fisher’s exact test was applied when ≥20% of the expected values of categorical data were <5. When significant differences were confirmed in the Kruskal–Wallis test, we additionally performed Dunn’s test to compare the ≥85 years group with the other group. When significant differences were confirmed in the chi-square test or Fisher’s exact test, we additionally performed a residual analysis to compare the ≥85 years group with the other group.

Overall survival (OS) and disease-specific survival (DSS) were expressed using the Kaplan–Meier method and assessed using the log-rank test and log-rank trend test. In addition, the Bonferroni adjustment was used to compare the two groups. Regarding OS and DSS, our research was designed to have an alpha error of 2.5% and power of 80% to perform the log-rank test with Bonferroni adjustment. As we estimated that the 5-year OS of patients aged ≥85 years was 60%, the presumed samples of patients aged ≥85 years were 45. For the Cox proportional hazard analysis, the number of explanatory variables was limited to one-tenth of the total number of deaths.

Propensity score matching was used to standardize the background characteristics among the groups. For propensity score matching for AEs, we selected the following covariates: sex, use of antithrombotic agents, renal disease, tumor diameter, en bloc resection, tumor location, and remnant stomach. We chose these covariates on the basis of previously reported risk factors for post-ESD bleeding (use of antithrombotic agents, renal disease, tumor diameter [31], en bloc resection, and tumor location [32]), perforation (tumor diameter and location [33]), post-ESD stricture (tumor diameter and location [34,35]), and aspiration pneumonia (remnant stomach [36]). For propensity score matching for OS, we chose the following patient characteristics as covariates: sex, American Society of Anesthesiologists (ASA) physical status, body mass index (BMI), comorbidities, and use of antithrombotic drugs.

The Kruskal–Wallis test, chi-square test, Fisher’s exact test, Dunn’s test, and residual analysis were performed using GraphPad Prism (version 6.07 for Windows; GraphPad Software, San Diego, CA, USA). The log-rank test, log-rank trend test, Cox proportional hazard analysis, and propensity score matching were performed using EZR (version 1.51; Jichi Medical University, Saitama, Japan). The *p*-values listed in each table are the results of the comparison between the three groups. Asterisks in each table indicate *p*-values that are <0.05, as the results of multiple comparisons with patients ≥85 years of age. A *p* value of <0.05 was considered statistically significant.

### 2.8. Study Approval

Informed consent was obtained through opt-out forms on our website. This study was approved by the ethical committee of Kitano Hospital (2205004, 11 May 2022) and was conducted in accordance with the Declaration of Helsinki.

## 3. Results

### 3.1. Patient Characteristics

**Figure 1:**
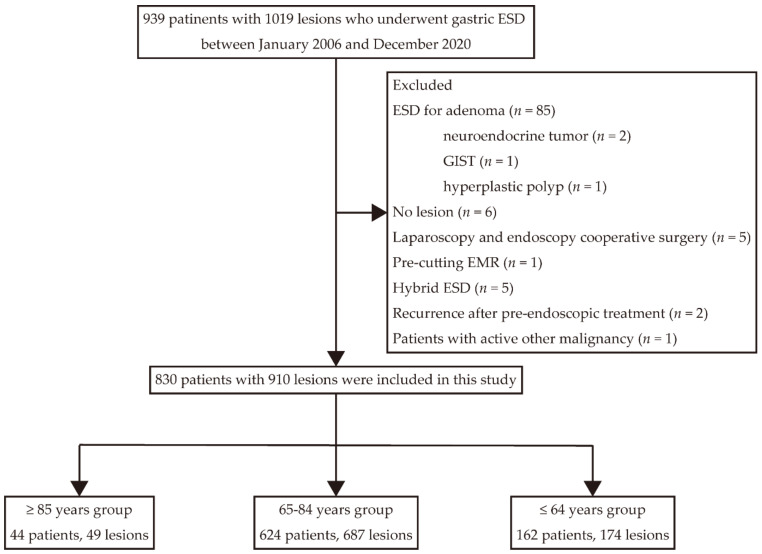
The flowchart of enrollment and selection of patients and lesions in this study.

A flowchart of the enrollment and selection of patients in this study is shown in Figure 1. A total of 939 patients with 1019 lesions underwent ESD between January 2006 and December 2020 at our hospital. After enrollment and selection, 44 patients with 49 lesions were in the ≥85 years group, 624 patients with 687 lesions were in the 65–84 years group, and 162 patients with 174 lesions were in the ≤64 years group.

The patients’ characteristics are listed in Table 1. The ASA physical status and Eastern Cooperative Oncology Group PS were significantly worse in the ≥85 years group (*p* < 0.001 and *p* < 0.001, respectively). The mean BMI was significantly lower in the ≥85 years group (*p* < 0.001). The proportion of patients with two or more comorbidities and the frequency of use of antithrombotic drugs were higher in the ≥85 years group (*p* < 0.001 and *p* < 0.001, respectively).

### 3.2. Pathological Characteristics

The pathological characteristics of the patients are summarized in Table 2. Macroscopic and histological types were significantly different among the three groups (*p* = 0.004 and *p* < 0.001, respectively). The tumor diameter was larger in the ≥85 years group than in the other groups (20.2 ± 9.5 mm, *p* = 0.025). However, no significant differences were found in tumor location, specimen diameter and depth, lymphatic invasion, vascular invasion, and ulcerative findings among the three groups.

### 3.3. Short-Term Outcomes

The short-term outcomes are presented in Table 3. The rate of en bloc resection in the ≥85 years group was 98.0% (48/49). The results did not differ among the three groups (*p* = 0.861). Moreover, the rate of curative resection in the ≥85 years group (87.8%, 43/49) was not significantly different from that in the other groups (*p* = 0.875). No significant differences in operation time and days of hospitalization were observed among the three groups.

### 3.4. Adverse Events

The original data on AEs are listed in Table 4. Although perforation was more frequently observed in the ≥85 years group (8.2%, *p* = 0.017), the total AE rate did not significantly differ among the three groups (*p* = 0.612). Aspiration pneumonia did not occur in the ≥85 years group, whereas one patient in the 65–84 years group had aspiration pneumonia (0.1%, 1/686). No ESD-related deaths occurred in the present study.

The odds ratios (ORs) of AEs after propensity score matching adjustment are shown in Table 5. AEs were not significantly more frequent in ≥85 years group (14.0%, 6/43) than in the 65–84 years group (23.2%, 10/43) (OR, 0.54; 95% confidence interval, 0.175–1.63; *p* = 0.272). Moreover, AEs were also not significantly more frequent in the ≥85 years group (12.9%, 4/31) than in the ≤64 years group (16.1%, 5/31) (OR, 0.77; 95% confidence interval, 0.186–3.19; *p* = 0.719).

### 3.5. Long-Term Outcomes

The long-term outcomes are summarized in Table 6. The median follow-up period in the ≥85 years group was 1151 days (range, 91–3893 days), which was significantly shorter than that in the other two groups (*p* < 0.001). The 3- and 5-year OS rates in the ≥85 years group were 85.7% and 61.9%, respectively, which were significantly worse than the rates in the other groups (*p* = 0.003 and *p* < 0.001, respectively). The proportion of patients who underwent additional surgery after non-curative resection was significantly smaller in the ≥85 years group than in the ≤64 years group (16.7%, 1/6) (*p* = 0.003). The characteristics and prognoses of the non-curative resection cases are summarized in Table S1.

**Figure 2:**
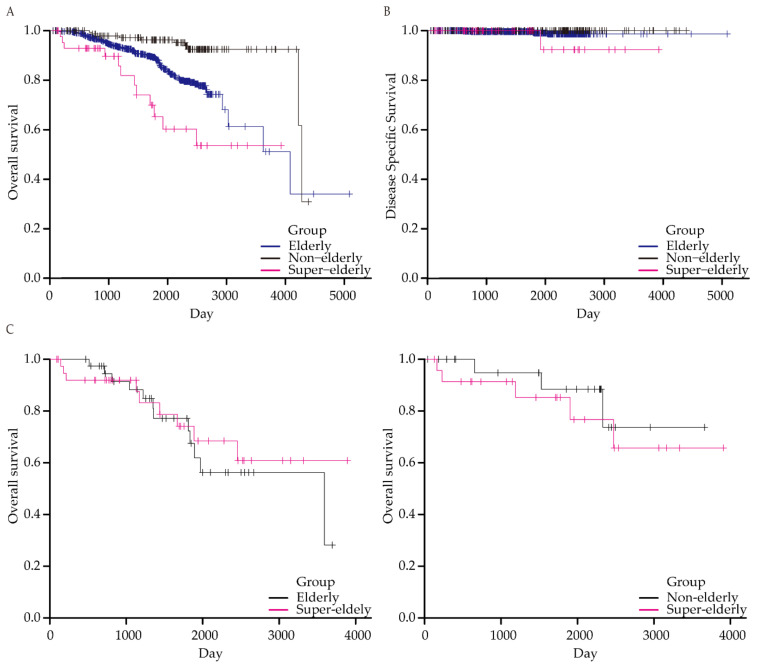
(**A**) Kaplan–Meier estimation of overall survival of the ≥85 years group, 65–84 years group, and ≤64 years group. (**B**) Kaplan–Meier estimation of disease specific survival of the ≥85 years group, 65–84 years group, and ≤64 years group. (**C**) Kaplan–Meier estimation of overall survival of the ≥85 years group and 65–84 years group (left) and ≥85 years group and ≤64 years group (right) after propensity score matching.

In total, 12 of the 44 (31.8%) patients ≥85 years died during the follow-up period. The OS was inferior among patients aged ≥85 years (*p* < 0.001, Figure 2A). However, only one patient in the ≥85 years group died of gastric cancer. Other patients died of colon cancer (*n* = 1), heart failure (*n* = 2), renal failure (*n* = 1), pneumonia (*n* = 3), or unknown causes (*n* = 4). Therefore, the DSS of the ≥85 years group was comparable, although a tendency was observed, with that of the other groups (*p* = 0.100, Figure 2B).

Subsequently, we calculated OS after propensity score matching. The OS in the ≥85 years group was not significantly different from that in the 65–84 years group (*n* = 39, *p* = 0.698) or ≤64 years group (*n* = 24, *p* = 0.516), although the sample sizes were relatively small (Figure 2C).

Finally, to determine the factors directly related to the survival of patients aged ≥85 years, we used the Cox proportional hazard model (Table 7). Among the chief clinical characteristics, pathological characteristics, short-term outcomes, and AEs, we found that a poor Geriatric Nutritional Risk Index (GNRI) was correlated with poor prognosis (HR, 0.89; 95% confidence interval, 0.83–0.95; *p* < 0.001).

## 4. Discussion

In this study, we confirmed that ESD is safe in patients aged ≥85 years (65–84 and ≤64 years). Although patients aged ≥85 years had worse OS, their DSS was not significantly different from those of patients in other age groups. Since poor GNRI was associated with poor prognosis of ESD for EGC in the ≥85 years group, the indications for ESD need to be decided carefully according to patient characteristics, especially nutritional status. Several reports on ESD for EGC in elderly patients have been published [6,13,37,38,39]. These studies defined the elderly population as those aged ≥ 70, 75, or 80 years. However, few reports have objectively defined a group of patients aged ≥85 years [11,12]. Therefore, our study is valuable in the era of an aging society. Our findings suggest that ESD can be safely performed in patients aged ≥85 years.

The total rate of AEs (81/909, 8.9%), including perforation (17/909, 1.9%), in this study was reasonable compared with that in previous studies performed in the general population (perforation rate, 2.2–4.5%) [6,40,41,42,43]. The results proved that our ESD procedures are valid and that our results are applicable to other institutions. Therefore, our study suggests that ESD is considerably safe in patients aged ≥85 years. The perforation rate was significantly higher in the ≥85 years group, as pointed out in a previous meta-analysis [44]. However, the total AE rate in the ≥85 years group was not significantly different from those in the other groups in this study. Although the rate of antithrombotic drug use was significantly higher in the ≥85 years group, no significant difference was observed in the rate of postoperative bleeding. None of the patients aged ≥85 years in this study had aspiration pneumonia, although a previous study reported an increased incidence of aspiration pneumonia in elderly patients [6,8,13,44]. 

In this study, the total rate of en bloc resection was 98.8% and that of curative resection was 89.4%. These were compatible with previous reports in the general population (en bloc resection rate, 92.7–99.2%; curative resection rate, 81.7–94.7%) [6,40,41,42,43], thus validating our results. In this context, the rates of en bloc resection (98.0%) and curative resection (87.8%) in the ≥85 years group were not significantly lower than those in the other groups. Our treatment results are comparable to or better than those in previous reports (en bloc resection rate, 91.6–97.9%; curative resection rate, 75.6–87.4%) [4,6,9,10,11,45]. These findings confirmed that the curability of ESD by EGC is independent of age.

In terms of long-term outcomes, the OS of the ≥85 years group was inferior to that of the other two groups. ASA physical status and PS were worse, and the rates of comorbidities and use of antithrombotic drugs were higher in the ≥85 years group. The BMI was lower in the ≥85 years group. Twelve patients in the ≥85 years group died during the follow-up. Such clinical characteristics could affect the OS of the ≥85 years group because propensity score matching adjustment for patient characteristics did not result in a significantly different OS in the ≥85 years group compared to the other groups.

In this study, one out of six patients with non-curative resection underwent additional surgery and the rate of additional surgery after non-curative resection was lower in the ≥85 years group than in the other groups. Among the five patients who did not undergo additional surgery, one patient died of gastric cancer. The estimated risk of lymph node metastasis in four patients who died due to other reasons or were alive without recurrence was 4.9% at most [25]. Therefore, the DSS did not significantly differ among the three groups. These findings suggest that additional surgery does not affect the prognosis of patients aged ≥85 years with non-curative resection in the case that the estimated risk of lymph node metastasis after non-curative resection is not high.

In our study, we found that the GNRI was correlated with the prognosis of ESD in patients aged ≥85 years. The GNRI is a simple index for evaluating the risk of nutrition-related morbidity and mortality [46]. A previous study also elucidated the values of GNRI in estimating prognosis [12]; hence, GNRI is a promising prognostic factor in patients aged ≥85 years. Our results suggest that we should carefully determine the indications for ESD in patients aged ≥85 years with poor nutritional status. In addition to the GNRI, previous reports have shown that renal dysfunction [9], cardiovascular disease [11], smoking, history of cancer of other organs, neutrophil-to-lymphocyte ratio, lymphovascular invasion, and Charlson comorbidity index [45] were risk factors for worse prognosis after ESD for EGC in patients aged ≥75 years. However, we did not find a correlation between prognosis and these factors partially because of the small sample size or missing data. Further accumulation of cases is needed to detect another prognostic factor in patients aged ≥85 years.

This study has some limitations. First, this was a single-center retrospective analysis. Therefore, our results may not be generalizable to other institutions. A selection bias was also present as patients who underwent ESD were expected to die of EGC progression and tolerate ESD procedures. Since we mostly excluded bedridden patients, those with dementia, or patients with severe impairment in ADL through the decision-making process, our results cannot be applied to every EGC patient aged ≥85 years. In addition, we included lesions in the remnant stomach or gastric tube in this study, which were sometimes excluded in other studies [9,11]. We aimed to include a real-world population of patients in this study. To adjust for risk factors for AEs, we performed propensity score matching in addition to raw analysis. Second, the sample size was relatively small. In this study, we could not include multiple explanatory variables in the Cox proportional hazard model because only 12 deaths after ESD occurred in the ≥85 years group. Therefore, for stronger statistical power, a multicenter study should be planned in the near future. Third, the learning curve and improvements in techniques may be important concerns because we analyzed the data from the beginning of ESD for EGC at our hospital. However, 20 (24 lesions) of the 44 patients (49 lesions) in the ≥85 years group underwent ESD during the earlier half of the investigation period (January 2006 to December 2014). Therefore, we believe that the learning curve and improvement in techniques did not substantially affect our results. Fourth, we could not follow up six patients aged ≥85 years who did not undergo ESD for EGC. If we could follow up those patients, we could compare them with patients aged ≥85 years who underwent ESD. However, this is difficult because we usually perform ESD when EGC is detected. In the case of colon cancer, a study has suggested that cancer resection has survival benefits in patients aged ≥85 years [47]. Further studies are needed in the field of EGC.

## 5. Conclusions

In conclusion, ESD can be safely performed in patients aged ≥85 years without an increased risk of AEs compared to other patients. The indications for ESD should be carefully decided considering not only the pathological characteristics but also the clinical characteristics of each patient, especially those with poor nutritional status.

## Figures and Tables

**Table 1:** Clinical characteristics of patients.

Clinical Characteristics	≥85 Years	65–84 Years	≤64 Years	*p* Value
Patients, *n*	44	624	162	
Lesions, *n*	49	687	174	
Median age, years (range)	86 (85–96)	74 (65–84) *	59 (35–64) *	<0.001
Sex (male/female)	30/14	456/168	129/33	0.155
Median ASA-PS (range)	2 (2–3)	2 (1–4)	2 (1–3) *	<0.001
ASA-PS (0–2/3–4)	32/12	441/183	136/26 *	0.003
Median ECOG-PS (range)	1 (1–3)	0 (0–3) *	0 (0–1) *	<0.001
ECOG-PS (0–1/2–4)	35/8	589/35 *	162/0 *	<0.001
BMI (mean ± SD), kg/m^2^	21.4 ± 2.2	23.3 ± 2.5 *	23.8 ± 2.9 *	<0.001
Comorbidities, *n* (%)				
Hypertension	33 (75.0)	375 (60.1) *	53 (32.7)	<0.001
Diabetes	8 (18.2)	157 (25.2)	19 (11.7)	0.001
Cardiovascular disease	8 (18.2)	134 (21.5)	15 (9.3)	0.002
Cerebrovascular disease	9 (20.5)	56 (9.0) *	2 (1.2) *	<0.001
Renal disease	3 (6.8)	23 (3.7)	2 (1.2)	0.096
Liver disease	3 (6.8)	41 (6.6)	6 (3.7)	0.383
Dyslipidemia	14 (31.8)	214 (34.3)	33 (20.4)	0.003
Having 2 or more comorbidities, *n* (%)	25 (56.8)	312 (50.0) *	22 (13.6) *	<0.001
Use of antithrombotic drugs, *n* (%)	25 (56.8)	186 (29.8)	13 (8.0) *	<0.001

* <0.05, as the results of multiple comparisons with ≥85 years.

**Table 2:** Pathological characteristics of the lesions.

Pathological Characteristic	≥85 Years	65–84 Years	≤64 Years	*p* Value
Lesions, *n*	49	686	174	
Location, *n* (%)				0.084
Upper	5 (10.2)	97 (14.1)	13 (7.5)	
Middle	17 (34.7)	275 (40.1)	82 (47.1)	
Lower	27 (55.1)	314 (45.8)	79 (45.4)	
Macroscopic type, *n* (%)				0.004
Elevated	31 (63.3)	353 (51.5)	70 (40.2)	
Flat	5 (10.2)	41 (6.0)	19 (10.9)	
Depressed	13 (26.5)	292 (42.6)	85 (48.9)	
Mean tumor diameter (mean ± SD), mm	20.2 ± 9.5	17.7 ± 9.2 *	15.3 ± 7.5 *	0.025
Mean resected specimen diameter (mean ± SD), mm	42.3 ± 13.5	42.4 ± 12.3	40.5 ± 12.6	0.660
Histology, *n* (%)				<0.001
Differentiated type	48 (98.0)	672 (98.0)	156 (89.7) *	
Undifferentiated type	1 (2.0)	12 (1.7)	17 (9.8) *	
Unknown	0 (0.0)	2 (0.3)	1 (0.6) *	
Depth, *n* (%)				0.551
M	45 (91.8)	599 (87.3)	157 (90.2)	
SM1	3 (6.1)	42 (6.1)	7 (4.0)	
SM2	1 (2.0)	42 (6.1)	8 (4.6)	
Unknown	0 (0.0)	3 (0.4)	2 (1.1)	
Lymphatic invasion, *n* (%)	1 (2.0)	28 (4.1)	4 (2.3)	0.599
Vascular invasion, *n* (%)	2 (4.1)	6 (0.9)	2 (1.1)	0.117
Ulcer, *n* (%)	2 (4.1)	19 (2.8)	4 (2.3)	0.748

* <0.05, as the results of multiple comparisons with ≥85 years.

**Table 3:** Short-term outcomes.

Short-Term Outcomes	≥85 Years	65–84 Years	≤64 Years	*p* Value
Patients, n	44	624	162	
Lesions, *n*	49	686	174	
En bloc resection, *n* (%)	48 (98.0)	678 (98.8)	172 (98.9)	0.861
eCura classification (A/B/C-1/C-2), *n*	40/3/3/3	589/24/7/66	151/6/1/16	0.058
Curative resection, *n* (%)	43 (87.8)	613 (89.4)	157 (90.2)	0.875
Operation time (mean ± SD), min	104.0 ± 61.1	100.7 ± 46.9	100.5 ± 47.7	0.924
Median days of hospitalization (range), day	9 (7–42)	9 (6–49)	8 (6–24)	0.212

**Table 4:** The original data on adverse events.

Adverse Events	≥85 Years	65–84 Years	≤64 Years	*p* Value
Lesions, *n*	49	686	174	
Total, *n* (%)	6 (12.2)	61 (8.9)	14 (8.0)	0.612
Postoperative bleeding, *n* (%)	2 (4.1)	49 (7.1)	10 (5.7)	0.605
Perforation, *n* (%)	4 (8.2)	10 (1.5) *	3 (1.7)	0.017
Stricture, *n* (%)	0 (0.0)	1 (0.1)	1 (0.6)	0.431
Aspiration pneumonia, *n* (%)	0 (0.0)	1 (0.1)	0 (0.0)	1.000
ESD-related death, *n* (%)	0 (0.0)	0 (0.0)	0 (0.0)	n/a

* <0.05, as the results of multiple comparisons with ≥85 years.

**Table 5:** Adverse events after propensity score matching adjustment.

Adverse Events	≥85 Years	65–84 Years	OR (95% CI)	*p* Value
Patients, *n*	43	43		
Adverse events, *n* (%)	6 (14.0)	10 (23.2)	0.535 (0.175–1.63)	0.272
	≥85 years	≤64 years	OR (95% CI)	*p* value
Patients, *n*	31	31		
Adverse events, *n* (%)	4 (12.9)	5 (16.1)	0.77 (0.186–3.19)	0.719

**Table 6:** Long-term outcomes.

Long-Term Outcomes	≥85 Years	65–84 Years	≤64 Years	*p* Value
Patients, *n*	44	624	162	
Median follow-up period (range), days	1151 (91–3893)	1793 (20–5053) *	2200.5 (25–4356) *	<0.001
3-year overall survival (%)	85.7	93.4	97.8 *	0.034
5-year overall survival (%)	61.9	83.0 *	95.1 *	<0.001
Additional surgery in non-curative resection, *n* (%)	1 (16.7)	36 (50.7)	15 (88.2) *	0.003
Total deaths, *n* (%)	12 (27.3)	100 (16.0)	10 (6.2) *	<0.001
Deaths caused by gastric cancer, *n* (%)	1 (2.3)	5 (0.8)	0 (0.0)	0.259

* <0.05, as the results of multiple comparisons with ≥85 years.

**Table 7:** Analysis of Cox proportional hazard model with prognosis in ≥85 years group.

Variables	HR (95% CI)	*p* Value
Age	0.86 (0.61–1.22)	0.401
Sex	1.36 (0.43–4.32)	0.598
ECOG-PS	2.31 (0.72–7.37)	0.159
BMI	0.85 (0.67–1.07)	0.166
Geriatric Nutritional Risk Index	0.89 (0.83–0.95)	<0.001
Having 2 or more comorbidities	1.29 (0.41–4.10)	0.664
Charlson Comorbidity Index	1.49 (0.87–2.58)	0.144
Use of antithrombotic drugs	2.6 (0.70–9.64)	0.153
Location		
M	0.37 (0.08–1.66)	0.195
L	0.55 (0.13–2.31)	0.410
Histological type	n/a	n/a
Macroscopic type		
Flat	0.00 (0.00-Inf)	0.998
Depressed	0.43 (0.09–1.97)	0.277
Curative resection	1.13 (0.30–4.19)	0.857
Operation time	0.99 (0.98–1.00)	0.136
Days of hospitalization	1.02 (0.96–1.09)	0.549
Adverse events	0.72 (0.09–5.58)	0.751

## Data Availability

Not applicable.

## Supplementary Materials

The following supporting information can be downloaded at: https://www.mdpi.com/article/10.3390/cancers14143311/s1, Table S1: Characters and prognosis of non-curative resection cases.
