# Supplementary material for: The Safety and Clinical Validity of Endoscopic Submucosal Dissection for Early Gastric Cancer in Patients Aged More Than 85 Years
Source: Cancers (Basel). 2022 Jul 7;14(14):3311. doi: 10.3390/cancers14143311 (PMC9323451; doi:10.3390/cancers14143311)
Supplement: Supplementary file 1 [file cancers-14-03311-s001.zip › cancers-1755841-supplementary.pdf]

Table S1. Characters and prognosis of non-curative resection cases.

Case	Age	Sex	*en-bloc* resection	Curability [24]	Cause of non-curative resecion	eCura system [25]	Additional surgery	Follow-up period (days)	Vital status	Cause of death
1	88	Male	Yes	C-2	VMX	1 (VMX)	Yes	3047	Alive	
2	85	Female	Yes	C-2	SM2, Ly1	4 (SM2, Ly1)	No	1887	Death	Gastric Cancer
3	86	Female	No	C-2	V1	2 (42 mm, V1)	No	1171	Death	Other
4	87	Female	Yes	C-1	HMX		No	1740	Death	Other
5	88	Male	Yes	C-1	HM1		No	3150	Alive	
6	85	Female	Yes	C-1	HM1		No	461	Alive	
